# Foxtail mosaic virus: A tool for gene function analysis in maize and other monocots

**DOI:** 10.1111/mpp.13330

**Published:** 2023-04-10

**Authors:** Bliss M. Beernink, Steven A. Whitham

**Affiliations:** ^1^ Department of Plant Pathology, Entomology, and Microbiology Iowa State University Ames Iowa USA; ^2^ Department of Biology University of Manitoba Winnipeg Manitoba Canada

**Keywords:** foxtail mosaic virus, maize, monocot, VIGE, VIGS, virus vector, VOX

## Abstract

Many plant viruses have been engineered into vectors for use in functional genomics studies, expression of heterologous proteins, and, most recently, gene editing applications. The use of viral vectors overcomes bottlenecks associated with mutagenesis and transgenesis approaches often implemented for analysis of gene function. There are several engineered viruses that are demonstrated or suggested to be useful in maize through proof‐of‐concept studies. However, foxtail mosaic virus (FoMV), which has a relatively broad host range, is emerging as a particularly useful virus for gene function studies in maize and other monocot crop or weed species. A few clones of FoMV have been independently engineered, and they have different features and capabilities for virus‐induced gene silencing (VIGS) and virus‐mediated overexpression (VOX) of proteins. In addition, FoMV can be used to deliver functional guide RNAs in maize and other plants expressing the Cas9 protein, demonstrating its potential utility in virus‐induced gene editing applications. There is a growing number of studies in which FoMV vectors are being applied for VIGS or VOX in maize and the vast majority of these are related to maize–microbe interactions. In this review, we highlight the biology and engineering of FoMV as well as its applications in maize–microbe interactions and more broadly in the context of the monocot functional genomics toolbox.

## Graphical abstract

A review of foxtail mosaic virus clones engineered for applications such as virus‐induced gene silencing, protein overexpression, gene editing, and virus‐induced flowering.

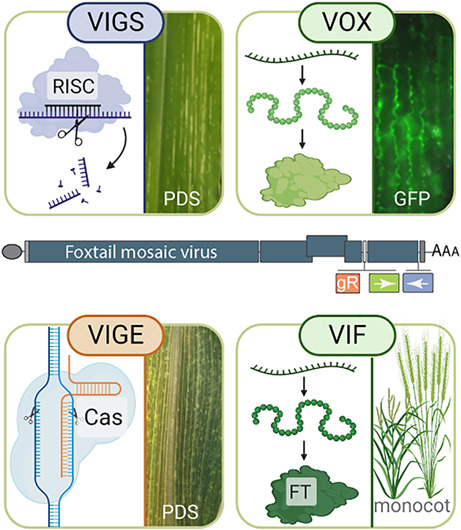

## 1 INTRODUCTION

Plant viruses are generally viewed unfavourably because their infections have a wide range of negative impacts on plant health. However, many plant viruses have been engineered to exploit their unique characteristics for positive aims. These engineered viruses have been characterized and utilized in plants for gene silencing, gene expression, and gene editing applications. The use of virus‐based vectors for gene function analysis in the field of plant biology has been exceptionally valuable for experiments in both model and non‐model organisms, and recent comprehensive reviews are available that describe many vectors and their applications (Abrahamien et al., 2020; Cody & Scholthof, 2019; Kant & Dasgupta, 2019; Khakhar & Voytas, 2021; Pasin et al., 2019; Rössner et al., 2022). With respect to host–pathogen interactions, recombinant viruses are used to silence host genes (virus‐induced gene silencing, VIGS), transiently express plant and pathogen genes (virus‐mediated overexpression, VOX), and silence pathogen genes (host‐induced gene silencing, HIGS). We anticipate that as virus‐induced gene editing (VIGE) applications improve there will be many examples of their use for modifying genes associated with host–pathogen interactions in the future (Gentzel et al., 2022).

*Zea mays* (maize) is a key cereal crop that is grown worldwide and is an important model species. There are many genetic and genomic resources available to facilitate gene function analyses, and over the years a growing number of virus‐based vectors have been reported to be useful in maize. At the present time, there are at least 11 different virus species that have been tested in maize for their utility in VIGS, VOX, and/or VIGE (Table 1). We also consider another use, virus‐induced flowering (VIF), which has not yet been demonstrated in maize, but is feasible in other monocots. VIF results from the transient overexpression of Flowering Locus T (FT) homologues in plants by means of a virus, and it is proposed to be of potential use to accelerate breeding programmes through the induction of flowering (Ayre et al., 2020; Yuan et al., 2020).

**TABLE 1:** Viral vectors developed for use in *Zea mays*.

Viral vector	Genus	VIGS	VOX	VIGE	VIF
Foxtail mosaic virus	*Potexvirus*	Mei et al. (2016) Liu et al. (2016)	Bouton et al. (2018) Mei et al. (2019)	Mei et al. (2019)	Yuan et al. (2020)^a^
Barley stripe mosaic virus	*Hordeivirus*	Jarugula et al. (2018)	Haupt et al. (2001)	Hu et al. (2019)	–
Sugarcane mosaic virus	*Potyvirus*	Chung et al. (2022)	Mei et al. (2019)	–	–
Maize dwarf mosaic virus	*Potyvirus*	Xie et al. (2021)	Xie et al. (2021)	–	–
Cucumber mosaic virus	*Cucumovirus*	Wang et al. (2016)	–	–	–
Maize rayado fino virus	*Marafivirus*	Mlotshwa et al. (2020)	–	–	–
Brome mosaic virus	*Bromovirus*	Ding et al. (2006)	–	–	–
Maize mosaic virus	*Nucleorhabdovirus*	–	Kanakala et al. (2022)	–	–
Barley yellow striate mosaic virus	*Cytorhabdovirus*	–	Gao et al. (2019)	–	–
Tobacco rattle virus	*Tobravirus*	Zhang et al. (2017)	–	–	–
Wheat streak mosaic virus	*Potyvirus*	–	Tatineni et al. (2010)		

Abbreviations: VIF, virus‐induced flowering; VIGE, virus‐induced gene editing; VIGS, virus‐induced gene silencing; VOX, virus‐mediated overexpression.

^a^ Used for monocots but not yet *Zea mays*.

Of the 11 virus species for which there is evidence that they may have utility as viral vectors in maize, each one has inherent advantages and disadvantages. Some viruses, such as cucumber mosaic virus (CMV), brome mosaic virus (BMV), and maize rayado fino virus (MRFV) (Ding et al., 2006; Mlotshwa et al., 2020; Wang et al., 2016), only have capacity to carry relatively small foreign inserts and therefore their use will probably be limited to VIGS and potentially VIGE (Willemsen & Zwart, 2019). Tobacco rattle virus (TRV), which is exceptionally useful in many dicots (Shi et al., 2021), was reported to cause VIGS of *phytoene desaturase* (*ZmPds*) in maize seedlings (Zhang et al., 2017), but the question of how well it can actually replicate and move systemically in maize has not been adequately addressed. Sugarcane mosaic virus (SCMV), maize dwarf mosaic virus (MDMV), and wheat streak mosaic virus (WSMV) are potyviruses that encode a large polyprotein. Protein expression in maize has been demonstrated via insertion of cloning sites that allow these viruses to express proteins from sequences that are cloned in frame with the viral polyprotein (Mei et al., 2019; Tatineni et al., 2010; Xie et al., 2021). Interestingly, gene fragments for VIGS applications can also be inserted into these positions as long as the open reading frame (ORF) is preserved (Chung et al., 2022; Xie et al., 2021). Moreover, it was shown that MDMV can be used to simultaneously express a protein and silence multiple target genes in maize plants (Xie et al., 2021). Because SCMV, MDMV, and WSMV encode large polyproteins, their genomes also serve as the sole viral messenger RNAs and therefore they do not produce shorter subgenomic messenger RNAs. The lack of subgenomic messenger RNA suggests that these viruses may not be useful for delivering guide RNAs for VIGE, but there may be strategies to overcome this limitation (Luo et al., 2021).

The viruses mentioned so far have single‐stranded, positive‐sense RNA genomes, and for these kinds of viruses the technology to produce infectious clones and manipulate them to accept the insertion of foreign sequences has been available since the 1980s (e.g., Ahlquist et al., 1984; French et al., 1986). Maize mosaic virus (MMV) and barley yellow striate mosaic virus (BYSMV) in contrast are negative (−)‐strand RNA viruses, and only recently has the ability to engineer infectious clones derived from them been demonstrated (Gao et al., 2019; Kanakala et al., 2022). These (−)‐strand RNA viruses are interesting because they have more stable insertions that are less susceptible to homologous recombination and spontaneous deletions, and they independently express multiple sequences, including ORFs, gene fragments, and guide RNA. As such, there is anticipation over their use for delivering clustered regularly interspaced short palindromic repeats (CRISPR) and CRISPR‐associated proteins (Cas) reagents as well as VIGS and VOX of multigenic metabolic pathways. If technical hurdles can be overcome related to initiating infections, which currently rely on inoculation into transgenic plant lines expressing the replication proteins and subsequent transfer of the resulting virions into experimental host plants by way of an insect vector, then these viruses may gain widespread use.

At the present time, the virus species that is most widely used in maize for gene function analyses is foxtail mosaic virus (FoMV). FoMV is becoming routinely used for VIGS and VOX, and it can deliver functional guide RNAs that can direct genome edits in maize plants expressing Cas9 protein, showing that it has potential for VIGE applications. For the remainder of this review we focus on FoMV biology and its engineering and use in gene function studies in maize and other monocots, and contributions of FoMV VIGS and VOX to investigating maize–microbe interactions are highlighted.

## 2 FoMV IS A POTEXVIRUS WITH A WIDE HOST RANGE

FoMV was first identified by the mild chlorotic mosaic symptoms it caused on the leaves of *Setaria viridis* (green foxtail) in a field in Kansas, United States (Paulsen & Niblett, 1977). FoMV is particularly interesting because it has a large experimental host range, infecting 56 monocot species and 35 dicot species, including numerous graminaceous species such as maize, *Hordeum vulgare* (barley), *Sorghum bicolor* (sorghum), *Setaria* spp. (millets), and *Triticum aestivum* (wheat) (Paulsen & Niblett, 1977). Despite its wide host range and ability to naturally infect weedy and crop hosts in the field (Paulsen & Niblett, 1977; Seifers et al., 1999), it has not been associated with major disease outbreaks or yield losses. *Foxtail mosaic virus* belongs to the genus *Potexvirus*, of which several species have been developed as viral vectors (Abrahamien et al., 2020). Potexviruses have been used as viral vectors due to their small but modifiable genomes, ability to spread systemically, and broad host range.

**FIGURE 1:**
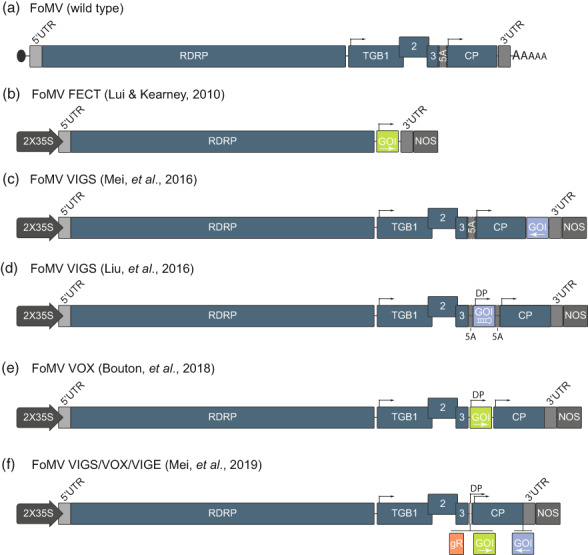
Schematic representations of foxtail mosaic virus (FoMV) and vectors that have been derived from it. (a) Wild‐type FoMV from left to right, the oval represents the 5′ 7‐methylguanosine cap structure, followed by a 5′ untranslated region (UTR), the RNA dependent RNA polymerase (RDRP), subgenomic promoter 1 (sgPro1) driving transcription of subgenomic RNA 1, the triple gene block proteins (TGB) 1, 2, and 3, a predicted open reading frame (ORF) 5A (5A) that is unnecessary for infection (Robertson et al., 2000), subgenomic promoter 2 (sgPro2) driving the transcription of subgenomic RNA 2, the coat protein (CP), a 3′ UTR that terminates at a polyA tail. (b) The FoMV FECT vector was developed as a virus overexpression (VOX) vector by replacing TGB1, 2, 3, and CP ORFs with a cloning site for foreign sequences expressed under control of sgPro1 (Liu & Kearney, 2010). The FoMV FECT vector consists of a 5′ UTR, RDRP, gene of interest (GOI) insertion site under control of sgPro1, and the 3′ UTR. (c) A virus‐induced gene silencing (VIGS) construct developed by Mei et al. (2016) by adding a multiple cloning site to the wild‐type FoMV genome immediately after the CP stop codon. Gene fragments are inserted at this location in the antisense orientation to induce silencing of endogenous plant genes. (d) FoMV VIGS vector developed by Liu et al. (2016) carries a duplicated sgPro2 (DP) that was inserted between TGB3 and the CP and preserves ORF 5A. Gene fragments are cloned as inverted repeats in the cloning site immediately following the DP. (e) The FoMV VOX vector (PV101) developed by Bouton et al. (2018) uses the duplicated sgPro2 promoter (DP) to drive expression of coding sequences inserted between TGB3 and CP. (f) Mei et al. (2019) developed and updated their FoMV vector to have the capacity for VIGS, VOX, and virus‐induced gene editing (VIGE). This version of FoMV is based on the Mei et al. (2016), and it includes a DP to drive expression of a GOI or produce functional single‐guide RNAs (gR) for CRISPR/Cas9 gene editing applications. All of the FoMV viral vector designs (b–f) are transcribed under the control of a 2× cauliflower mosaic virus 35S promoter (35S) and the nopaline synthase (NOS) terminator, and the grey arrows located along the genomes represent the positions of the sgPro1, sgPro2, and DPs.

The genome of FoMV was first sequenced and published in 1991 and revised in 2008 after infectious full‐length clones were generated and sequenced (Bancroft et al., 1991; Bruun‐Rasmussen et al., 2008). Like other potexviruses, FoMV has a single‐stranded, positive‐sense RNA genome that is 6.2 kilobases (kb) in length. It encodes five different proteins from five ORFs, and possesses a 5′ 7‐methylguanosine cap structure and 3′ polyadenylated tail (Abou Haidar & Gellatly, 1999) (Figure 1a). ORF 1 produces a 152 kDa replication protein with methyltransferase, helicase, and RNA‐dependent RNA polymerase (RDRP) domains. The replication protein produces new copies of the viral genome and also generates two subgenomic messenger RNAs from viral subgenomic promoters 1 and 2 (sgPro1 and sgPro2). ORFs 2, 3, and 4 are collectively known as the triple gene block; the proteins (TGB1, 2, and 3) are expressed from sgPro1 and are involved in suppressing plant antiviral defences as well as providing movement functions. Lastly, ORF 5 produces the coat protein (CP) from sgPro2, which is necessary for virion assembly and long‐distance movement (Candresse et al., 2012). Unlike other potexviruses, an ORF 5A was also identified in the FoMV genome (Figure 1a), but mutations disrupting the start codon showed that it is dispensable for infection and its disruption had no impact on viral replication and fitness (Mei et al., 2019; Robertson et al., 2000).

## 3 ENGINEERING FoMV FOR VIGS, VOX, AND VIGE

Potexviruses have been good candidates for development into viral expression vectors due to their ability to accept a duplicated subgenomic promoter and lack of theoretical virion size constraints due to being rod‐shaped, filamentous viruses (Willemsen & Zwart, 2019). Potato virus X (PVX) is the archetypal potexvirus that was first engineered for transient gene expression in plants (Chapman et al., 1992). Initial PVX expression vector designs replaced the CP with the coding sequence of the marker protein, β‐glucuronidase (GUS). While GUS was highly expressed, this strategy prevented systemic movement of the virus (Chapman et al., 1992). A second strategy duplicated the CP promoter (sgPro2) and inserted it between the TGB3 and CP ORFs. The GUS coding sequence was fused to the duplicated sgPro2 promoter, and this recombinant PVX successfully expressed GUS systemically and set a precedent for designing potexvirus expression vectors (Chapman et al., 1992).

Subsequently, many potexviruses have been engineered as expression vectors, including bamboo mosaic virus (BaMV) (Chen et al., 2017; Lin et al., 1996; Liou et al., 2014), Cymbidium mosaic virus (CymMV) (Hsieh et al., 2013), cassava common mosaic virus (CsCMV) (Tuo et al., 2021), Plantago asiatica mosaic virus (PlAMV) (Minato et al., 2014), narcissus mosaic virus (NMV) (Zhang et al., 2013), Alternanthera mosaic virus (AltMV) (Lim et al., 2010), Zygocactus X virus (ZVX) (Koenig et al., 2006), pepino mosaic virus (PepMV) (Abrahamian et al., 2021; Sempere et al., 2011), and FoMV (Bouton et al., 2018; Liu et al., 2016; Mei et al., 2016, 2019). Of these nine potexvirus species, only FoMV, BaMV, PlAMV, and CymMV can infect monocot plants. FoMV's large host range combined with its ability to systemically infect many monocots, while often inducing relatively mild mosaic symptoms, makes it an ideal candidate for viral vector development for use in important crop plants.

The first infectious clone of FoMV was generated by Robertson et al. (2000), and it was utilized for site‐directed mutagenesis to test the functions of the predicted viral ORFs. This clone was later modified by Liu and Kearney (2010) to generate what the authors have named the “FECT” expression vector. In FECT, the FoMV genome is expressed under transcriptional control of a cauliflower mosaic virus (CaMV) 35S promoter and nopaline synthase (NOS) terminator in a binary T‐DNA plasmid. FECT is not capable of local or systemic movement because the TGB and CP ORFs were removed (Figure 1b). Foreign sequences are cloned after sgPro1, which drives their expression. FECT is inoculated into leaf tissues via *Agrobacterium* infiltration (agroinfiltration), and because this virus is incapable of movement, protein expression occurs only at the site of infiltration. Local expression of the foreign protein is dramatically enhanced by co‐expression of the tomato bushy stunt virus (TBSV) p19 gene silencing suppressor. This approach works well for high levels of protein expression in plants such as *Nicotiana benthamiana* that support efficient agroinfiltration and replication of FoMV amplicons in the presence of p19.

Subsequently, a few different variations of fully functional FoMV clones have been independently developed and tested for systemic VIGS, VOX, and/or VIGE. Mei et al. (2016) constructed a clone derived from FoMV isolate PV139 that can only be used for VIGS (Figure 1c). The FoMV genome is placed between the CaMV 35S promoter and the NOS terminator, and the genome was modified to include cleavage sites for the XbaI and XhoI restriction enzymes immediately after the CP stop codon. This position enabled the insertion of foreign sequences for VIGS applications, but it is not possible to express proteins. This FoMV vector was inoculated directly into maize by DNA particle bombardment, and the infected plants were directly assessed for VIGS phenotypes or the infected tissues were collected, stored, and used as a source of inoculum to infect more experimental plants by rub‐inoculation. Via the FoMV VIGS vector, silencing of maize *pds* in sweetcorn (Golden x Bantam) and in the inbred B73 genotype was achieved. Expression of *lesion mimic 22* (*les22*), *iojap*, and *brown midrib 3* was also silenced in sweetcorn, but only down‐regulation of *les22* and *iojap* resulted in the expected phenotype. The FoMV isolate was also tested for its ability to infect a panel of 10 maize inbred lines, and all of them except Mo17 were susceptible. These results suggest that FoMV can be used for VIGS in a broad range of maize germplasm. The inability of the FoMV clone to infect Mo17 maize is consistent with prior work showing that Mo17 has resistance to FoMV through a quantitative trait controlled by eight loci (Ji et al., 2010).

Liu et al. (2016) developed a VIGS vector derived from the original infectious clone produced by Robertson et al. (2000) (Figure 1d). The FoMV genome was placed between the 2× CaMV 35S promoter and the NOS terminator in a T‐DNA plasmid derived from pBin19. Subsequently, the sgPro2 was duplicated in a way that preserved the ORF 5A, and cleavage sites for the HpaI, MluI, XhoI, and AscI restriction enzymes were inserted just after the duplicated sgPro2. To generate inoculum, this clone is agroinfiltrated into *N*. *benthamiana*, and then the infected leaves are used to inoculate monocot plant species: barley, wheat, or *Setaria italica* (foxtail millet). A 200‐nucleotide (nt) sequence targeting barley *pds* was cloned into this virus, but it induced only mild VIGS. However, when short, inverted repeat sequences were cloned at this position, robust silencing of target genes was observed in barley (*pds*, *Magnesium chelatase subunit 1*), foxtail millet (*pds*, *Chloroplatos alterados 1*(*CLA1*), *Isopentenyl/dimethylallyl diphosphate synthase*), and wheat (*pds*, *CLA1*). The use of this vector for VIGS in maize was not reported.

Bouton et al. (2018) developed an FoMV VOX vector named PV101 that was also derived from FoMV isolate PV139 (Figure 1e). PV101 carries a 101‐nt duplication of the CP promoter (sgPro2) to drive expression of genes of interest, and the dispensable ORF 5A is disrupted. An interesting aspect in the construction of this vector is that PV101 was synthesized based on passaging of FoMV through wheat seven times followed by sequencing. This passaged virus had 83‐nt differences from the prototype version of the infectious clone. The wheat‐optimized PV101 genome containing the 101‐nt sgPro2 duplication followed by a multiple cloning site was synthesized and inserted into a binary T‐DNA plasmid under transcriptional control of the CaMV 35S promoter and the NOS terminator. PV101 is first agroinoculated into *N*. *benthamiana* to generate an inoculum that can be used to infect wheat, maize, or other monocots. PV101 was used to express the 600 amino acid GUSPlus protein in wheat and maize, demonstrating its ability to spread systemically as it carries an 1800‐nt insert. Furthermore, the authors demonstrated that expression of pathogen effector proteins was feasible via the delivery of the fungal pathogen *Stagonospora nodorum* ToxA protein, which induced cell death as expected in wheat.

Mei et al. (2019) modified their original FoMV VIGS vector (Mei et al., 2016) by moving its genome into a binary T‐DNA plasmid under transcriptional control of the CaMV 35S promoter and NOS terminator to enable agroinfiltration. In addition, they also duplicated the CP sgPro2 along with the addition of a second multiple cloning site (Figure 1f). This clone retains the original VIGS cloning site immediately after the CP stop codon, and thus it is possible to use this vector for simultaneous VOX and VIGS, although that has not been demonstrated. The duplicated CP promoter incorporated mutations to disrupt the start codon of ORF 5A, thus eliminating it and reducing sequence redundancy with the wild‐type sgPro2. This clone was used to express green fluorescent protein (*GFP*) and bialaphos resistance (*BAR*) in maize plants where the expected phenotypes of green fluorescence and resistance to glufosinate herbicide, respectively, were observed.

## 4 METHODS FOR INOCULATING FoMV VIRAL VECTORS

For viral vectors to become broadly utilized for functional genomics and gene editing delivery systems, it is necessary to have efficient and accessible plant inoculation methods. Current methods for launching viral infections from infectious clones include in vitro transcription followed by vascular puncture inoculation (VPI), rub‐inoculation, or particle bombardment to introduce infectious RNA transcripts into plants. Alternatively, DNA constructs encoding the viral genomes under plant promoters can be introduced using VPI, rub‐inoculation, particle bombardment, and agroinfiltration. Each method has advantages and limitations associated with it. If the viruses are mechanically transmissible, the inoculated leaves and/or systemically infected leaves of a few inoculated plants can be stored for later use to inoculate many experimental plants using rub‐inoculations or VPI (Mei & Whitham, 2018; Redinbaugh et al., 2001; Scholthof, 1999). However, some viruses are not mechanically transmissible, and so the rub‐inoculation and VPI methods using infected leaf sap are not technically feasible in these cases (e.g., MMV and BYSMV). For FoMV, all these approaches are feasible, which provides flexibility in generating and saving inoculum for later uses.

In particular, *Agrobacterium*‐based methods that deliver engineered viral genomes into the host cells are the simplest and least expensive methods available for viral vector delivery (Vaghchhipawala et al., 2011; Zhang et al., 2017). While agroinfiltration works well in many dicot species, it is difficult and very inefficient in most monocot species, and for this reason it is often preferred to initiate infections in *N*. *benthamiana* to generate the inoculum for experiments with the monocot plant of interest. This approach requires that *N*. *benthamiana* can also be a host for the engineered virus, which is possible for some viruses (e.g., FoMV, barley stripe mosaic virus [BSMV], and BMV) but not others (e.g., SCMV, MDMV). To bypass the need to first use *N*. *benthamiana* for generating FoMV inoculum, Beernink et al. (2021) developed a protocol for direct agroinoculation of maize with FoMV or SCMV. This method was inspired by classic work with infectious clones of maize streak virus, a geminivirus, that could be inoculated into maize by injecting the *Agrobacterium* strains into the whorl of seedlings 2–3 mm above the shoot apical meristem (Grimsley et al., 1986). This method can be applied for both FoMV VIGS and VOX applications, but there may be a negative correlation between insert size and inoculation efficiency (Beernink et al., 2021).

## 5 EXPERIMENTAL DESIGN CONSIDERATIONS

In addition to inoculation methods, we provide some key considerations to aid in the design and interpretation of experiments using FoMV systems. At the most basic level, it is necessary to determine if the required maize genotype(s) is susceptible to FoMV or not (e.g., Mo17). If there are options, then selection of the particular FoMV vector could be a consideration. At this time, we are not aware that the different vectors (Figure 1) have been tested directly against one another, but as discussed in sections 3 and 6.1, it appears that PV101 (Bouton et al., 2018) has been used successfully for expression of relatively large proteins or fusion proteins, which has not yet been demonstrated for other FoMV clones.

As with most viral systems, there is inherent variability in FoMV VIGS and VOX experiments. There are a number of reasons for this that include inoculation efficiency, not all cells are uniformly infected, host genotype, insert sequence, and insert stability. When using their agroinjection method in maize seedlings, Beernink et al. (2021) found that infection rates for recombinant FoMV carrying 329‐ and 313‐nt gene fragments were similar to the empty vector but much higher than FoMV‐GFP, which carries a 711‐nt insertion. Additionally, the FoMV infection rate varied among 10 susceptible maize genotypes (Beernink et al., 2021).

The effectiveness of VIGS and VOX can also be altered by the stability of the insert. For example, a 300‐nt insert targeting sorghum phytoene desaturase (*SbPds*) was stably maintained at 21 days postinoculation (dpi) in 72%–90% of plants, but a 300‐nt insert targeting ubiquitin (*SbUb*) was stably maintained in only 36%–45% of the plants in sorghum genotype BTx623 (Bredow et al., 2022). Interestingly, in the sorghum genotype BTx430, the *SbPds* gene fragment was stably maintained in FoMV in 100% of the plants screened, and the *SbUB* fragment was stable in only 12%–25% of the plants. These data show that the insert sequence and host genotype can influence insert stability. However, time after inoculation is also critical. The retention of the *SbPds* fragment was similar at 14, 21, and 28 dpi, and the *SbUb* insert was stable at 14 dpi but became increasingly unstable at 21 and 28 dpi. These data from sorghum are consistent with prior results from maize using a *ZmPds* insert (Mei et al., 2016). In plants that were inoculated at 7 days after sowing, the *ZmPds* insert was stably retained in leaves 4–6 but as later leaves developed, the insert in FoMV became increasingly unstable. By leaf 9, *ZmPds* was beginning to be deleted and was fully retained in 75% of the plants and in leaves 12–13 the insert was intact in approximately 25% of the plants. The level of gene silencing measured by reverse transcription‐quantitative PCR (RT‐qPCR) correlated with the loss of insert (Mei et al., 2016).

Based on our experience with the vectors shown in Figure 1a,f, we propose the following recommendations as a guide for effectively using FoMV for VIGS, VOX, and VIGE. Preliminary testing is required to ensure compatibility of FoMV with host plants and to establish infection rates under the experimental conditions. Due to inherent variability, three independent replications with at least six to 10 plants each are recommended to produce statistically meaningful outcomes. Controls should include the empty vector construct and a mock treatment to demonstrate that FoMV itself is not influencing the phenotype of interest. Due to concerns about stability, the integrity of the insert should be confirmed by RT‐PCR in the tissues that are being used for phenotyping and assessing target gene silencing or heterologous protein expression.

## 6 APPLICATIONS OF FoMV VECTORS IN UNDERSTANDING OF MAIZE–MICROBE INTERACTIONS

To date, the primary application of FoMV vectors has occurred in topics related to maize–microbe interactions (Table 2). Here, we highlight their application in some of the studies focused on maize–microbe interactions that have benefited from the availability of these resources for investigating the functions of both host and pathogen genes. Plants can recognize the presence of pathogens through the action of pattern‐recognition receptors that activate pattern‐triggered immunity (PTI) in response to conserved molecular features, such as flagellin (flg22 peptide, bacteria) or chitin (fungi) (Yu et al., 2017). Activation of PTI is accompanied by a variety of changes, including reactive oxygen species (ROS) burst, callose deposition, and increased expression of defence genes. Successful pathogens secrete effectors that inhibit PTI by targeting different proteins involved in regulating or mediating it (Toruño et al., 2016). Resistance proteins recognize the presence of effectors, either directly or indirectly, and activate effector‐triggered immune responses (ETI) that often result in hypersensitive cell death (HR) (Cui et al., 2015). Much remains to be learned about the regulation of PTI, ETI, resistance protein function, and pathogen effector functions in maize, and VIGS and VOX approaches are contributing key information that is helping to advance understanding of these various facets of maize–microbe interactions.

**TABLE 2:** Foxtail mosaic virus used as a viral vector in plants.

Plant species	VIGS	VOX	VIGE	VIF	Target genes
Proof of concept in maize					
*Zea mays*	Mei et al. (2016)	–	–	–	*ZmPDS, Zmles22, Zmij, Zmbm3*
*Z. mays*	Burkhow et al. (2018)	–	–	–	*ZmPDS*
*Z. mays*, *Nicotiana benthamiana, Triticum aestivum*	–	Bouton et al. (2018)	–	–	*GFP, GUSPlus*
*Z. mays, Setaria viridis, N. benthamiana*	–	Mei et al. (2019)	Mei et al. (2019)	–	*GFP, BAR, sgNbPDS, sgSvCA2, sgZmHKT1*
*Z. mays, N. benthamiana*	–	–	Beernink et al. (2022)	–	*sgNbPDS, ‐AtFT, ‐AttRNAIle, sgZmHKT1, ‐AtFT, ‐ZCN8, ‐ZCN16, ‐ZCN19, ‐AttRNAIle*
Maize–microbe interactions					
*Z. mays*	Ma et al. (2018)	–	–	–	*ZmAFP1, ZmAFP2*
*Z. mays*	Han et al. (2019)	–	–	–	*ZmKWL1*
*Z. mays*	Tanaka et al. (2019)	–	–	–	*ZmTTK2, ZmTTK3*
*Z. mays*	–	Darino et al. (2021)	–	–	*UmJsi1*
*Z. mays*	Dressano et al. (2020)				*ZmIRR*
*Z. mays*	–	Jiao et al. (2021)	–	–	*MCMVp31, MCMVp7a, MCMVRTD, GFP*
*Z. mays (Rp1‐D21)*	Murphree et al. (2020)	–	–	–	*ZmSGT1, ZmRAR1, ZmHSP90, LOX9, ZmVPS37, ZmHCT, ZmCCoAOMT, Zm PGH1, ZmPk1b, ZmQCR7, ZmSL11, ZmIQM3*
*Z. mays (Rp1‐D21)*	Karre et al. (2021)				*ZmEIL1, ZmMYB83*
*Z. mays*	–	Navarrete et al. (2021)	–	–	*UmTay1, UmMer1*
*Z. mays*	–	Saado et al. (2022)	–	–	*UmRIP1*
*Z. mays*	Xu et al. (2022)	–	–	–	*ZmTGL*
*Z. mays*	Yu et al. (2022)	–	–	–	*ZmPDS, ZmFLR1/2, ZmFLR3*
Other species					
*N. benthamiana*	–	Liu & Kearney (2010)	–	–	*GFP*
*Hordeum vulgare, T. aestivum, Setaria italica*	Liu et al. (2016)	–	–	–	*HvPDS, HvChlH, TaPDS, TaCLA1, SiPDS, SiCLA1, SiIspH*
*N. benthamiana*	Chang et al. (2017)	–	–	–	*NbRDR6*
*N. benthamiana*	–	Zhang et al. (2020)	Zhang et al. (2020)	–	*sgNbPDS, Cas9, p19*
*H. vulgare, N. benthamiana*	–	Alonso et al. (2020)	–	–	*UhAVR1, GFP*
*Phalaenopsis aphrodite*	Kuo et al. (2021)	–	–	–	*PaAGO5a, PaAGOb*
*Alopecurus myosuroides*	–	Mellado‐Sanchez et al. (2020)	–	–	*GFP, BAR*
*Panicum miliaceum, T. aestivum*	–	–	–	Yuan et al. (2020)	*NtFT, SFT, Hd3a, AtFT*
*S. italica*	Dangol et al. (2021)	–	–	–	*SiTDC1*
*Sorghum bicolor*	Bredow et al. (2022)	–	–	–	*SbPDS, SbUb, SbRLCK1, SbRLCK2, SbRLCK3*
*N. benthamiana*	–	Prakash et al. (2023)	–	–	TuMV *6K2*
*Panicum virgatum*	Tiedge et al. (2022)	–	–	–	*PvChlD, PvChlI, PvPDS*

Abbreviations: VIF, virus‐induced flowering; VIGE, virus‐induced gene editing; VIGS, virus‐induced gene silencing; VOX, virus‐mediated overexpression.

### 6.1 Use of FoMV vectors in *Ustilago maydis–*maize interactions

Based on several publications, it is clear that the *U. maydis* research community has been particularly active in their adoption of FoMV vectors to explore the functions of *U*. *maydis* effectors. In three different studies, FoMV VIGS was used to silence the expression of maize genes encoding proteins that are targeted by *U*. *maydis* or *Sporisorium reilianum* effectors (Han et al., 2019; Ma et al., 2018; Tanaka et al., 2019) (Table 2). In these studies, 300‐nt target gene fragments selected using the Sol Genomics Network VIGS tool (vigs.solgenomics.net) were cloned in the antisense orientation into the Mei et al. (2016) FoMV VIGS vector. Silencing of target genes was confirmed by RT‐qPCR, and biological assays determining effects on fungal growth and plant defences were performed.

FoMV has also been used to ectopically express *U*. *maydis* effectors as fusion proteins with epitope tags. In three different expression studies, the Bouton et al. (2018) PV101 vector was used to express effectors lacking their signal peptides that were fused to the myc or HA epitopes (Darino et al., 2021; Navarette et al., 2021; Saado et al., 2022) (Table 2). The effector fusions were co‐expressed with the p19 protein, which is a suppressor of RNA silencing that promotes accumulation of the recombinant viruses. The mCherry protein was also co‐expressed with p19 and the effector fusion in two of the studies, which provides a non‐destructive reporter on virus accumulation and spread. Ectopic expression of the effectors enabled analysis of their roles in promoting cell death or suppressing host defences.

### 6.2 Use of FoMV vectors to investigate genes involved in maize immunity

FoMV VIGS has been used to investigate the functions of maize genes from the perspectives of resistance protein signalling and control of basal defence responses. Yu et al. (2022) used the Mei et al. (2019) FoMV vector to silence three homologues of the maize Feronia‐like receptor (ZmFLR) (Table 2). One construct targeted *ZmFLR1* and *ZmFLR2*, and a second one targeted *ZmFLR3*. *ZmFLR1* and *ZmFLR2* had to be co‐silenced due to high sequence homology. To separately silence *ZmFLR1/2* and *ZmFLR3*, it was necessary to target the least conserved region encoding the transmembrane domain. VIGS of *ZmFLR1*, *2* and *3* reduced their expression by approximately 65%–70%, and ROS production was dramatically reduced in response to flg22 and chitin application to leaf discs. Plants in which *ZmFLR1/2* or *ZmFLR3* were silenced were challenged with four different fungal pathogens, and disease severity increased for all providing evidence that the ZmFLRs have important roles in maize antifungal immune responses.

In the largest‐scale VIGS study in maize to date, Murphree et al. (2020) used the Mei et al. (2016) FoMV VIGS vector to target 12 different maize genes to test if their silencing affected the *Rp1‐D21* lesion mimic phenotype (Table 2). Target genes were chosen for this study based on the following criteria: (i) homologues in other species are required for the functions of nucleotide‐binding site (NBS) leucine‐rich repeats (LRR) resistance proteins, such as Rp1‐D; (ii) previously shown to be required for the *Rp1‐D21* autoactive HR phenotype; or (iii) loci affecting *Rp1‐D21* function identified through genome‐wide association studies (GWAS). For three of the genes (*LOX9*, *Pk1b*, *Sl11*), there was no successful silencing. The remaining nine genes, plus *Rp1‐D21*, were silenced between approximately 1.6‐ and 17‐fold. Of these, four genes suppressed HR when silenced (*Rp1‐D21*, *HSP90*, *VPS37*, *IQM3*), demonstrating that they are required for *Rp1‐D21* function, three genes enhanced the *Rp1‐D21*‐HR when silenced (*HCT*, *CCoAOMT*, *SGT1*), and for another three genes, silencing them had no effect on the phenotype (*PGH1*, *QCR7*, *RAR1*). These data show that VIGS can be used to validate gene function predictions based on functions of homologous genes in other species and from GWAS.

### 6.3 Use of FoMV to investigate maize–virus interactions

Maize chlorotic mottle virus (MCMV) causes maize lethal necrosis when it co‐infects maize along with unrelated viruses, such as potyviruses like SCMV (Redinbaugh & Stewart, 2018). Jiao et al. (2021) were interested in the molecular mechanisms underlying the pathogenicity of MCMV, and found some preliminary evidence that its 31 kDa protein (p31) was a major pathogenicity determinant. They expressed individual MCMV proteins p31, p7a, and the readthrough domain (RTD) or a GFP control fused to a 3× FLAG tag from the PV101 FoMV vector in B73 maize seedlings. FoMV expressing p31 or the RTD induced necrotic lesions on maize leaves but p7a and the GFP control did not. These data demonstrated that the RTD portion of p31 is responsible for the necrosis induced by MCMV infection. Subsequently, they showed the FoMV expressing p31 suppresses salicylic acid (SA) production as well as the expression of PR genes when co‐inoculated with MCMV. These results showed that p31 suppresses the SA‐mediated defence responses induced by MCMV.

Xu et al. (2022) used the Liu et al. (2016) FoMV vector to silence *ZmTGL*, which codes for the production of triacylglycerol (Table 2). *ZmTGL* was identified as interacting with SCMV's helper component proteinase (HC‐Pro) through protein pull‐down and tandem mass spectrometry. Silencing *ZmTGL1* reduced its mRNA transcripts by 50% and resulted in a 2–3‐fold greater accumulation of SCMV. This silencing phenotype is consistent with a role for ZmTGL1 in reducing the accumulation of SCMV HC‐Pro, which is a silencing suppressor required for efficient replication of SCMV.

## 7 FoMV AND VIGE

Engineering viruses to deliver gene editing components has been a rapidly expanding area of research. The use of viral vectors overcomes bottlenecks associated with traditional transgenesis methods that are needed to introduce gene editing reagents into plants (Yin et al., 2017). Virus‐based delivery of gene editing reagents can potentially open access to gene editing or enhance gene editing efficiency in many plant species without the need to go through the processes of transformation and regeneration (Scholthof et al., 1996).

Targeted gene editing technologies have revolutionized genetics over the past decade. Meganucleases, zinc finger nucleases (ZFNs), and transcription activator‐like effector nucleases (TALENs) are all genome editing platforms with a high level of target specificity, but they are limited by challenges in modifying that specificity (Voytas & Gao, 2014). Clustered regularly interspaced short palindromic repeat (CRISPR) arrays and CRISPR‐associated proteins (e.g., Cas9) have been harnessed to activate, suppress, delete, and add new target genes in the genomes of many organisms (Belhaj et al., 2015; Bortesi & Fischer, 2015; Doudna & Charpentier, 2014; Pennisi, 2013). CRISPR‐based genome editing technologies continue to be developed and improved for new applications as well as increased efficiency and target specificity. The utility of these systems is the ease with which they can be reprogrammed through the delivery of specific single‐guide RNAs (sgRNAs). Several plant viruses with positive‐sense RNA genomes that had previously been used for VIGS and/or VOX were demonstrated to also deliver sgRNAs systemically and induce edits in host plants that express Cas proteins. Some of the first viral systems established to deliver sgRNAs and successfully validate gene editing include tobacco rattle virus (TRV) (Ali, Abul‐faraj, Li, et al., 2015; Ali, Abul‐faraj, Piatek, et al., 2015), tobacco mosaic virus (TMV) (Cody et al., 2017), and pea early browning virus (PEBV) (Ali et al., 2018). Gene editing as a result of virus‐delivered sgRNAs predominantly occurs in somatic cells, but some viruses expressing sgRNA have been shown to efficiently induce heritable genome edits, such as TRV and PVX in *N*. *benthamiana* and BSMV in wheat (Beernink et al., 2022; Ellison et al., 2020; Li et al., 2021; Uranga et al., 2021). In the case of TRV, the efficiency of inducing heritable genome edits is augmented significantly if a mobile RNA sequence, such as *Arabidopsis* FT, is fused to the sgRNA (Beernink et al., 2022; Ellison et al., 2020). However, for BSMV, the addition of RNA mobility sequences hinders the ability of the virus to induce heritable genome edits in wheat (Li et al., 2021).

Functional sgRNA delivery was explored using FoMV in *N*. *benthamiana*, maize, and *S*. *viridis* (Beernink et al., 2022; Mei et al., 2019). FoMV clones carrying sgRNA were able to induce somatic genome edits in the *Pds* gene of *N*. *benthamiana* plants expressing Cas9. The induced mutations were small insertions and deletions (indels), and they occurred in leaves and flowers over the course of plant development (Mei et al., 2019). However, the level of mutation was not sufficient to cause the photobleaching phenotype expected for *Pds* loss of function and heritable mutations were not observed, which is in contrast to TRV, PVX, and BSMV in *N*. *benthamiana* (Beernink et al., 2022). Interestingly, the frequency of mutations induced by FoMV expressing sgRNA targeting *NbPds* could be dramatically enhanced by co‐infection with turnip mosaic virus (TuMV), which is a potyvirus that promotes greater accumulation of FoMV and other viruses through the action of its silencing suppressor. Unfortunately, the boost in FoMV accumulation in the presence of TuMV is lethal to the plant (Mei et al., 2019). FoMV expressing sgRNA was also able to induce genome edits in Cas9‐expressing *S*. *viridis* and maize plants (Mei et al., 2019). In maize, the frequency of genome editing in leaves was relatively low compared to *N*. *benthamiana* and *S*. *viridis*, and heritable mutations were also not observed (Beernink et al., 2022). Like in *Cas9 N*. *benthamiana*, it was possible to enhance FoMV‐induced gene editing by co‐infection with a potyvirus, SCMV, but co‐infected *Cas9* maize plants developed severe disease symptoms and were mostly sterile if they survived to flowering (Mei et al., 2019). The potential for RNA mobility sequences to enhance FoMV‐induced gene editing was investigated in *Cas9 N*. *benthamiana* and *Cas9* maize. There was evidence that the presence of RNA mobility sequences can enhance FoMV‐induced somatic gene editing in both species, but it was not sufficient to promote germline mutations (Beernink et al., 2022).

## 8 CONCLUSIONS

FoMV‐based vectors have become valuable research tools that are used to silence and overexpress genes involved in maize–microbe interactions. Candidate genes identified as interacting with pathogen effectors, homologues of key immunity‐related genes from other plant species, and maize GWAS, transcriptomics, proteomics, and gene regulatory network studies can be rapidly investigated. We expect that FoMV VIGS can be used as a complementary strategy with stable maize mutants identified as transposon insertions or generated using RNAi or CRISPR‐Cas mutagenesis. For example, many candidate genes can be readily screened by VIGS and the outcomes can be used to prioritize genes for which it is desirable to obtain stable mutant or knockdown alleles for more in‐depth research.

Moreover, the studies presented show that FoMV VIGS and VOX can be used successfully to investigate resistance gene function, PTI, and maize–fungus and maize–virus interactions. We also anticipate that it will be useful to investigate maize–bacteria interactions, although this is yet to be demonstrated. For example, Bredow et al. (2022) showed that FoMV VIGS of receptor‐like cytoplasmic kinases in sorghum suppressed basal immune responses rendering the plants more susceptible to bacterial pathogens. Similarly, FoMV VOX of fungal effector proteins and viral proteins has been very useful for exploring maize–microbe interactions, and we would anticipate that this would be the case for effectors encoded by bacteria, nematodes, and insects as well. So far, VIGS and VOX have been demonstrated to work in leaves and switchgrass roots (Tiedge et al., 2022), and so it will be interesting to see if these approaches can be applied to other organs in the future. In addition, it will be interesting to determine if FoMV can be used as a vector for host‐induced gene silencing (HIGS) to knockdown the expression of genes of pathogens as they attempt to infect plants in which FoMV carrying fragments of pathogen genes are replicating, as has been shown for other viruses (Hu et al., 2020; McCaghey et al., 2021; Nowara et al., 2010; Panwar et al., 2013; Yin et al., 2015).

It is exciting to see that FoMV‐based resources and their corresponding protocols are being adopted successfully by many laboratories. While FoMV has been applied mainly for research in maize–microbe interactions at this time, we anticipate that it will be useful in studying the functions of genes involved in other aspects of maize biology. A major rationale for engineering FoMV for VIGS and VOX applications was its reportedly broad host range, particularly in monocots (Scofield & Nelson, 2009), and several recent publications suggest that FoMV is meeting expectations (Table 2). In addition to maize, FoMV has been demonstrated to be useful for VIGS, VOX, VIGE, or VIF in eight other monocot species, including seven grasses and one orchid species. The list of grass species includes five grain crop species (*H*. *vulgare*, *S*. *italica*, *T*. *aestivum*, *Panicum* *milliaceum*, and *S*. *bicolor*), one biomass crop species (*Panicum* *virgatum*), and two weed species (*S*. *viridis* and *Alopecurus* *myosuroides*). Success in these organisms coupled with the broad experimental host range of FoMV suggests that there are many additional species that currently lack gene function analysis or plant transformation technologies for which FoMV‐based vectors can be utilized.

## CONFLICT OF INTEREST STATEMENT

The authors declare no conflict of interest.

## Data Availability

Data sharing is not applicable to this article as no new data were created.
